# A synthetic biology approach to assemble and reboot clinically relevant *Pseudomonas aeruginosa* tailed phages

**DOI:** 10.1128/spectrum.02897-23

**Published:** 2024-01-31

**Authors:** Thomas Ipoutcha, Ratanachat Racharaks, Stefanie Huttelmaier, Cole J. Wilson, Egon A. Ozer, Erica M. Hartmann

**Affiliations:** 1 Department of Civil and Environmental Engineering, Northwestern University, Evanston, Illinois, USA; 2 Division of Infectious Diseases, Department of Medicine, Feinberg School of Medicine, Northwestern University, Chicago, Illinois, USA; 3 Center for Synthetic Biology, Northwestern University, Evanston, Illinois, USA; University at Albany, Albany, New York, USA

**Keywords:** phage therapy, synthetic biology, *Pseudomonas aeruginosa*, phage reboot

## Abstract

The rise in the frequency of antibiotic resistance has made bacterial infections, specifically *Pseudomonas aeruginosa*, a cause for greater concern. Phage therapy is a promising solution that uses naturally isolated phages to treat bacterial infections. Ecological limitations, which stipulate a discrete host range and the inevitable evolution of resistance, may be overcome through a better understanding of phage biology and the utilization of engineered phages. In this study, we developed a synthetic biology approach to construct tailed phages that naturally target clinically relevant strains of *Pseudomonas aeruginosa*. As proof of concept, we successfully cloned and assembled the JG024 and DMS3 phage genomes in yeast using transformation-associated recombination cloning and rebooted these two phage genomes in two different strains of *P. aeruginosa*. We identified factors that affected phage reboot efficiency like the phage species or the presence of antiviral defense systems in the bacterial strain. We have successfully extended this method to two other phage species and observed that the method enables the reboot of phages that are naturally unable to infect the strain used for reboot. This research represents a critical step toward the construction of clinically relevant, engineered *P. aeruginosa* phages.

**IMPORTANCE:**

*Pseudomonas aeruginosa* is a bacterium responsible for severe infections and a common major complication in cystic fibrosis. The use of antibiotics to treat bacterial infections has become increasingly difficult as antibiotic resistance has become more prevalent. Phage therapy is an alternative solution that is already being used in some European countries, but its use is limited by the narrow host range due to the phage receptor specificity, the presence of antiviral defense systems in the bacterial strain, and the possible emergence of phage resistance. In this study, we demonstrate the use of a synthetic biology approach to construct and reboot clinically relevant *P. aeruginosa* tailed phages. This method enables a significant expansion of possibilities through the construction of engineered phages for therapy applications.

## INTRODUCTION

*Pseudomonas aeruginosa* (PA) is a Gram-negative bacterium responsible for 51,000 infections with 2,700 deaths in the US every year (1) and approximately 559,000 deaths globally in 2019 (2). PA is also a common complication of cystic fibrosis (CF), with 80% of CF patients developing PA infection (3) and causing chronic infection in 41% of un-transplanted adults with CF (4). Antimicrobial resistance of PA infections has become an increased concern (1, 5). This is particularly the case in low- and middle-income countries where multidrug-resistant bacteria are more prevalent (6).

Phage therapy is a promising alternative for treating infections (7–10). In 2022, the World Health Organization included it as a priority to fight antibiotic resistance, which is classified as a major concern over the next 5–10 years (11). For PA, phage therapy with naturally isolated phages has been developed successfully (7), but the use of phage is limited by the phage specificity, which depends on the presence of phage receptors and defense systems [e.g., CRISPR systems and restriction-modification (RM)]. Furthermore, even in sensitive strains, resistance is likely to arise through phage receptor mutations (12). To avoid resistance, alternatives like phage cocktails and/or combinations of phages and antibiotics have been used (13, 14). Unfortunately, not all combinations are synergistic (15), and a greater understanding of phage-bacteria interactions is needed to choose optimal combinations.

Phage engineering has the potential to improve phage therapy efficiency and avoid phage resistance (16). The intent is to design phage therapy specific to the bacterial strain considering the phage receptor and the presence of antiviral defense systems to make the application of phage safer and more effective. Phage engineering encompasses a variety of applications, including inhibiting replication and changing the cargo carried. For example, phagemids, which consist of a phage capsid carrying a plasmid and cannot replicate in nature, have been designed in response to the need for safe technology. Engineered phagemids have been used to deliver a CRISPR system to antimicrobial-resistant strains of *Staphylococcus aureus* (17) or deliver antimicrobial enzymes (18). While this approach is promising, it is restricted to well-characterized phages like M13 in *Escherichia coli* or P1 in PA (19).

Phages can also be modified to be more suitable for therapeutic applications, e.g., to change phage host range by altering the phage tail fiber (17, 20) or adding anti-CRISPR to bypass adaptative defense systems (21). These modifications are often performed using homologous recombination in the host bacteria (10). However, those methods are restricted to small rearrangements of non-essential proteins and are limited by the recombination efficiency (22). Other platforms have been used for both phage construction and production of particles, i.e., “reboot.” For example, Cheng et al. (23) used *Escherichia coli* to assemble, edit, and reboot a large panel of phages, including PA phages, to target Gram-negative bacteria, but as acknowledged in the study, no clinically relevant tailed phages have been rebooted and the methodology does not work for all phages. This limitation has been discussed in several papers (24, 25) and could be explained by the presence of toxic proteins encoded in the phage genome and subsequently expressed in *E. coli* (26, 27).

To avoid the limitations associated with working in *E. coli*, it is possible to separate phage engineering into two steps: (i) assembly of the synthetic genome and (ii) reboot of phage particles with a synthetic genome. One well-known platform for the construction and engineering of various bacterial and viral genomes is the yeast *Saccharomyces cerevisiae* (28–30). In contrast to *E. coli*, prokaryotic DNA, including toxic molecules that could be encoded by phages, is rarely expressed in yeast and does not impact yeast fitness (31). Yeast has been used for this purpose to clone or construct synthetic phage genomes, changing tail fiber specificity (24). While yeast is useful for producing synthetic phage genomes, they are incapable of producing phage particles, i.e., performing “reboot.” For Gram-negative phages, reboot is still performed in *E. coli*, which again restricts the method to only certain phages. Recently, some *S. aureus* and *Enterococcus faecalis* phages were constructed in yeast and rebooted directly in *S. aureus* (25). Furthermore, *Pseudomonas* phage vB_PaeP_PE3 has been cloned and engineered in yeast to construct a reduced phage genome, which was successfully rebooted in PAO1 (32). Although vB_PaeP_PE3 is part of the Autographiviridae family and cannot infect clinically relevant PA strains (32, 33), this study demonstrates the feasibility of genome manipulation in yeast. It remains, however, unclear how generalizable the results are and whether all PA phages are amenable to this process.

**Fig 1:**
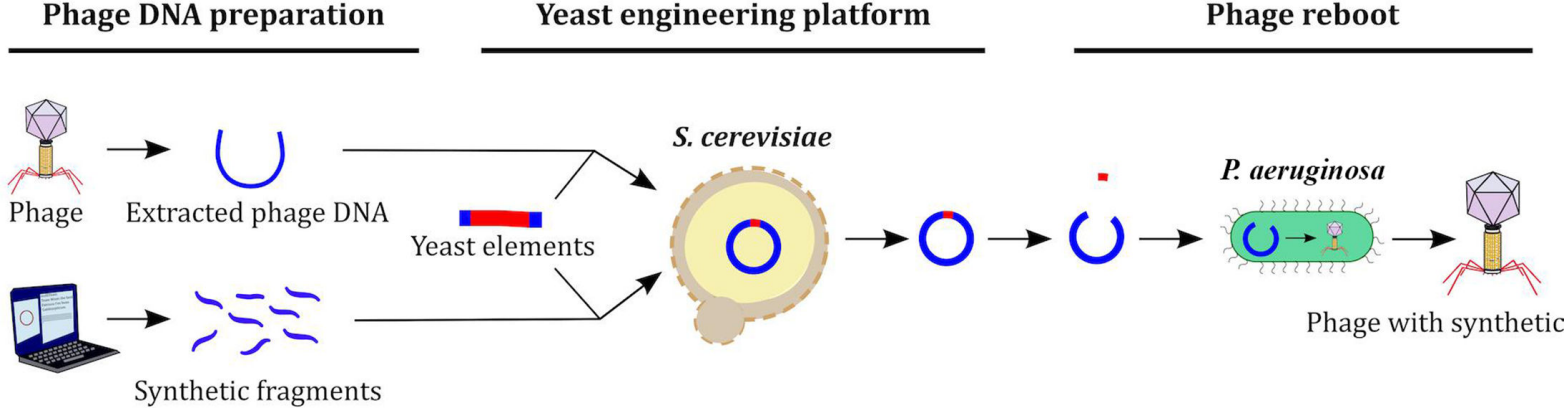
Schematic representation of the experimental procedure. Using direct extraction of the phage genome or the construction of overlapping fragments amplified by PCR, we were able to clone or construct the phage genome in yeast and maintain it using yeast elements. Next, extraction of yeast DNA and digestion by restriction enzymes allowed us to obtain full-length phage DNA that is free from yeast elements. Finally, PA transformation permitted us to obtain rebooted phage particles.

Engineering phage genomes in yeast enables large and diverse modifications, but the resulting genomes still need to be rebooted. In the current study, we examine the use of yeast for genome engineering, followed by reboot using PA. We focus on addressing limitations in the reboot process by examining JG024, a member of the genus *Pbunavirus*, which are lytic phages that infect numerous clinically relevant PA strains and are thus considered candidates for phage therapy (34–38). JG024 (39) was extensively studied for this application in combination with antibiotics (40). We develop a methodology for the construction of synthetic phage particles using transformation-associated recombination (TAR) cloning with yeast followed by rebooting the phage DNA into *P. aeruginosa* to produce viable phage particles (Fig. 1). Comparing reboot success between different phages in PA led us to identify factors that limit phage reboot, including phage-specific characteristics and host antiviral defense systems. This work represents the first time PA phages of high interest for phage therapy applications are successfully rebooted from synthetic genomes produced in yeast.

## RESULTS

### Corroboration of a circular permuted JG024 genome

**Fig 2:**
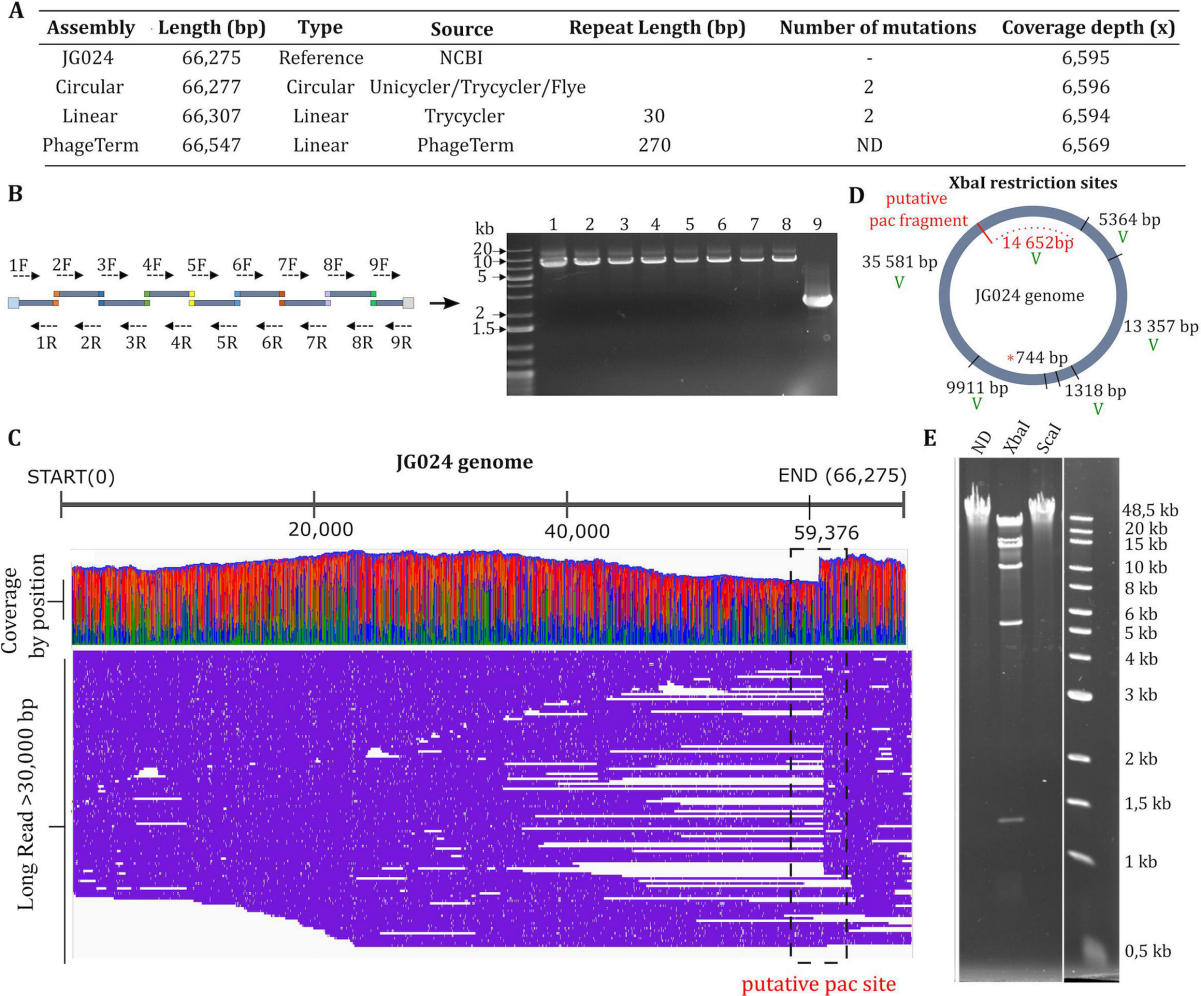
Analysis of JG024 genome. (A) Overview of assembly results compared to the reference genome from NCBI; assembly was performed using Unicycler, Trycycler, Flye, and PhageTerm. (B) Visual representation of overlapping fragments used for the amplification of the full JG024 genome. (C) Coverage by position (with colors representing base calls: A, green; T, red; G, orange; and C, blue) and location of long reads (>30,000 bp) mapped to the JG024 reference genome using IGV. (D) Representation of the expected digestion sites from a circular JG024 genome using XbaI; fragments marked with a green “V” are observed on the corresponding agarose gel. (E) Agarose gel of XbaI digestion; ND undigested JG024 genome; XbaI, digestion of JG024 genome with XbaI; and ScaI digestion of JG024 genome with ScaI.

To enable the development of a successful cloning and reboot strategy, it is critical to characterize the genome of the phage in question. We thus sequenced the genome of our JG024 (Fig. 2A), revealing both conserved structural features and population-level heterogeneity. Compared to the published JG024 genome (66,275 bp) (39), we observed two insertions, one G at position 29,132 (in 52% of short reads) and one A at position 55,007 (in 97% of short reads, 337th amino acid position of ORF F358_gp71). We confirmed these two mutations by Sanger sequencing, indicating that they are not artifacts of the sequencing process but rather reflect population-level heterogeneity in the phage.

Hybrid assembly generated a circular molecule of 66,277 bp (Fig. 2A) using three approaches. In addition, one (Trycycler) produced a linear assembly of 66,307 bp, which was identified by CheckV to contain direct terminal repeats (DTRs) of 30 bp. In contrast, PhageTerm identified DTRs of 270 bp, resulting in a linear genome of 66,547 bp. To verify the presence of either the 30 or 270 bp DTRs in the two linear assemblies, “primer walking” was used (Fig. S1A). For both linear assemblies, DTRs were not identified, as there was no termination of the sequence or decrease in signal intensity after the proposed DTR sequence. Instead, the sequence continued beyond the DTR suggesting a continuous sequence akin to a circular assembly. Only one known phage genome structure could result in circular assembly of phage dsDNA: circular permuted genomes. In this case, a packaging site (*pac* site) is usually recognized by a phage protein to initiate DNA packaging, but the terminase has poor specificity and nonspecific headful cleavage happens when the capsid is full, resulting in the presence of phage genome sizes ranging from 98% to 110% of the reference phage genome (41). Our assembly suggests that the JG024 genome is a circularly permuted genome and that the phage uses a headful packaging strategy.

To corroborate this hypothesis experimentally, we successfully amplified the entire viral genome using primers to generate nine overlapping fragments (Fig. 2B). Although JG024 was previously identified to have a linear genome through exonuclease *Bal31* digestion (39), the amplification of the entire viral genome using overlapping fragments suggests that JG024 has no physical ends as suggested by the circularized long-read assembly (Fig. 2A). Furthermore, as each successfully amplified fragment must originate from at least some virion DNA molecules that contain the entire length of the fragment, this is consistent with the idea of a circularly permuted genome. Additionally, if we observe the global distribution of all long reads greater than 30,000 bp obtained from our sequencing efforts (Fig. 2C), we observed a decrease in coverage depth between positions 50,000 and 60,000. We also see that a larger proportion (36/161) of reads start at position 59,376 (±5 bp). This could be the packaging series initiation site (*pac* sequence) recognized by the phage terminase protein for DNA packaging.

As previously described for P22, SPP1, and P1 phages (41), restriction of circular permuted genomes results in fragments that would be predicted from a circular molecule, with an additional *pac* fragment sometimes observed. Concerning JG024, we observed that the genome was not sensitive to three enzymes (ScaI), suggesting that the DNA is methylated (Fig. 2E; Fig. S1C). We further did not observe digestion with NdeI or BsaI (data not shown), despite the presence of predicted digestion sites. Using XbaI, we observed that the restriction digest profile corresponds to a circular permuted genome (Fig. 2E) and disagrees with what would be predicted for a linear genome (Fig. S1B and D); this is in contrast to previous conclusions in the study by Garbe et al. (39), which predicted that the genome was linear despite incongruous results from SacII digestion. Their conclusion was based on a linear map of the JG024 genome, but their result could correspond to a circular digestion profile (8.5 + 21.7 + 35.9 kb). In addition to the bands predicted from a circular assembly, we observed a restriction band around 15,000 bp that does not correspond to a band predicted from a linear profile. This band matches the predicted *pac* fragment starting from the putative *pac* site at position 59,376 bp (Fig. 2C through E).

Together, these data suggest that the JG024 genome is circular permuted. This knowledge is important for designing the cloning strategy in yeast and will guide us to use linear-linear recombination to assemble and maintain the JG024 phage genome.

### Assessment of chloroform sensitivity and other parameters to improve reboot efficiency

**Fig 3:**
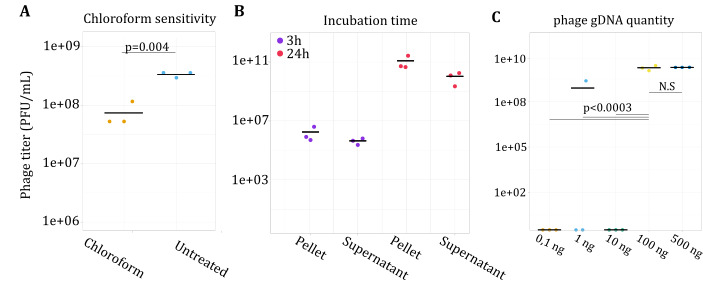
Phage yield (in titer) following infection or reboot under different experimental conditions. (A) PA14 was infected with JG024 with and without chloroform treatment to assess sensitivity. Chloroform was found to have a significant effect on phage titer (analysis of variance, ANOVA; *P* = 0.004) and decreased the phage titer. (B) Following infection, PA14 was allowed to recover 3 or 24 h, and phages were collected either from the cell pellet (C) or supernatant (S). A total of 100 ng of gDNA from this recovered phage solution was then electroporated into PA14 to determine the impact of recovery time (3 vs 24 h) and phage release (C vs S) on yield. The phage titer of the phages found in the supernatant was 11-fold higher than the phages released from chloroform extraction (ANOVA; *P* = 0.01). C- JG024 was rebooted using different starting amounts of phage gDNA in PA14. The quantity of JG024 gDNA was found to have a significant effect on the phage titer (ANOVA; *P* = 0.0003) as only gDNA quantities of at least 100 ng resulted in consistent plaques. There was no significant difference in phage titer between 100 and 500 ng of gDNA (ANOVA; *P* > 0.05) with phage titer reaching an average of 1.8 × 10^10^ PFU/mL for both DNA quantities.

To optimize the reboot protocol and avoid issues linked to low reboot or transformation efficiency, we assessed how different parameters affected phage titer. Chloroform is often used during phage production to destroy bacterial cells and release phage particles in the bacterial lysate (42). As chloroform affects 30% of tailed phages (43), we assessed the effect of chloroform on JG024. JG024 phage lysate was treated with chloroform before infecting PA14, and JG024 plaques were then enumerated using the double agar method. Chloroform significantly affected phage titer (*P* = 0.004), which decreased 4.57-fold (78.2% reduction) compared to the untreated phage lysate [3.4 × 10^8^ plaque-forming unit (PFU)/mL] (Fig. 3A), indicating that JG024 is sensitive to chloroform.

To investigate if JG024 phages are well released from PA14 cells during the rebooting process, JG024 gDNA (25 and 100 ng) was electroporated into electrocompetent PA14 cells and incubated for either 3 or 24 h. After incubation, the cell suspension was pelleted and the supernatant was assessed directly for PFU to quantify the phages released naturally from phage-mediated cell lysis. The remaining cell pellet was washed three times with LB media, treated with chloroform, and assessed for PFUs to quantify the phages released primarily from chloroform treatment. The 3-h incubation was sufficient to observe PFUs but only in two of three replicates when 25 ng of gDNA was used. Extending the recovery time significantly increased the number of PFUs (*P* = 0.0001) and resulted in consistent PFU formation in all replicates. This observation agrees with expectations for lytic phage in a sensitive bacterial culture. Furthermore, phage particles were found in the same quantity in the supernatant or bacterial pellet after chloroform release (Fig. 3B). We further attempted to reboot JG024 using a higher quantity of JG024 gDNA. However, there was no significant difference in phage titer between 100 and 500 ng of gDNA (*P* > 0.05) with phage titer reaching an average of 1.8 × 10^10^ PFU/mL for both DNA quantities (Fig. 3; Supplemental figures and text).

We finally investigated the effect of different PA strains on JG024 reboot efficiency. Using strain PAO1, we obtained a greater number of PFUs and more consistent results compared to PA14 (Fig. S2D). These results indicate that specific host-strain factors are critical to phage infection and replication. Other transformation parameters, such as wash buffer (300 mM sucrose vs 1 mM MgSO_4_), MgSO_4_ concentration after electroporation (0, 1, and 10 mM MgSO_4_), and electroporation voltage (1.8, 2.2, and 2.5 kV), were also tested (Fig. S2A through C). The buffer had a significant effect on the phage titer with the use of MgSO_4_ resulting in higher phage titer than sucrose (*P* = 0.002). The phage titer from 2.2 kV was higher than the phage titer from 1.8 kV (*P* = 0.02). These data suggest that a reboot protocol without the use of chloroform and using optimized buffer and electrophoresis conditions can improve reboot efficiency. We also observed that a high concentration of JG024 phage DNA and PA strain-specific characteristics can increase reboot success.

### Successful cloning and construction of JG024 genome in yeast

**Fig 4:**
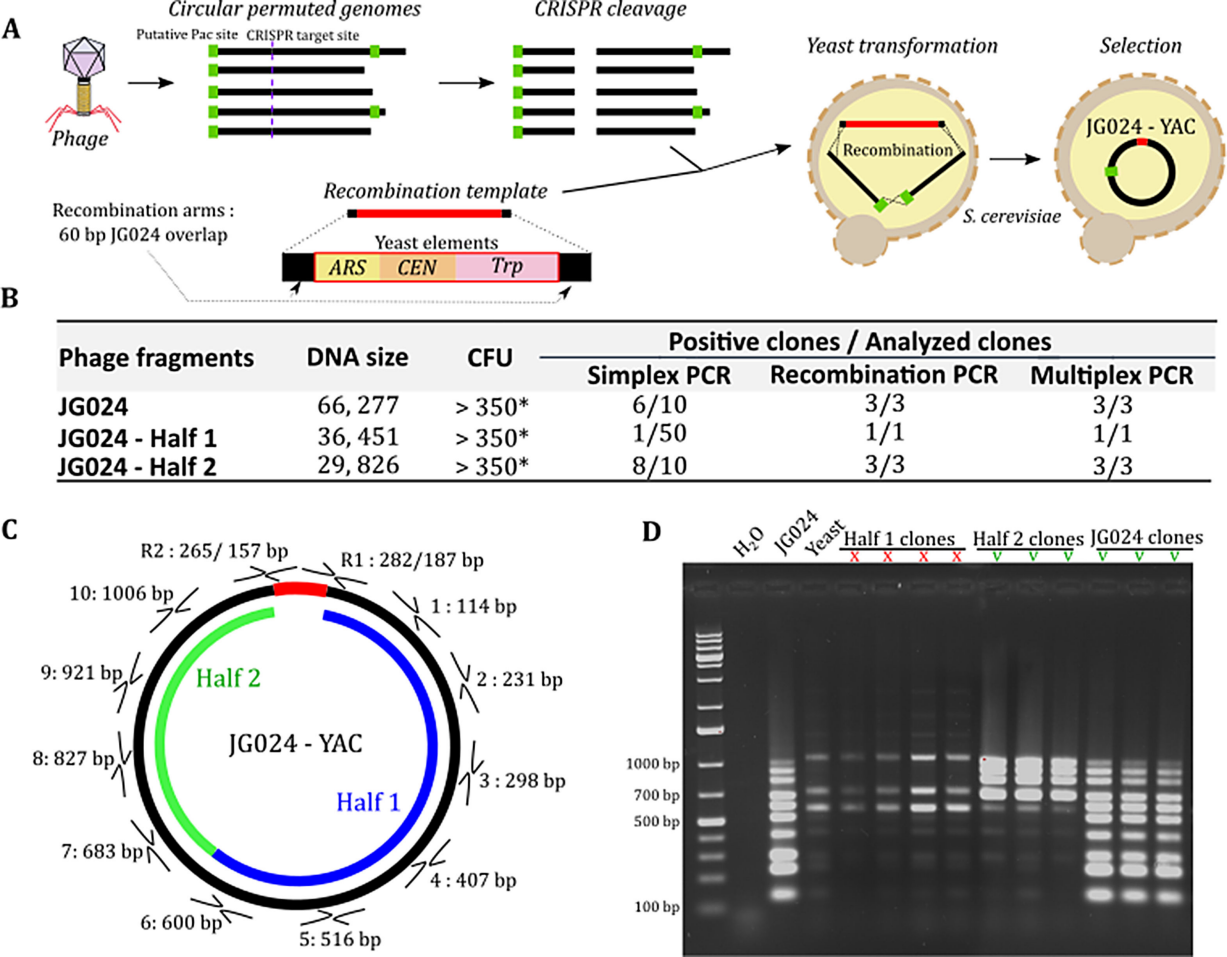
Reassembly of the JG024 genome in yeast using TAR cloning. (A) Schematic of TAR-cloning procedure. (B) Cloning efficiency of the entire JG024 genome and two smaller parts in yeast. Simplex PCR consists of one PCR that amplifies a single region of the genome. Recombination PCR involves the amplification of recombination scars, and multiplex PCR uses a set of several primers to amplify multiple regions around the phage genome (in this case, 10). (C) Representation of the three batches of PCR done to validate the cloning of the phage genome in yeast. (D) Example agarose gel of multiplex PCR products performed to validate phage genome integrity in clones. Expected bands were produced from the intact genome and Half 2, but Half 1 only yielded bands corresponding to untransformed yeast controls.

Based on previous work for the cloning of full bacterial and viral genomes (29, 44–46), we chose the yeast *S. cerevisiae* VL6-48N as a platform to clone and replicate JG024 DNA (Fig. 4A). TAR cloning (47) has been used extensively for the isolation and production of large genomic fragments from a variety of organisms.

For cloning JG024 in yeast, we used the full-length JG024 genome and a recombination template flanked by 60 bp of homology (recombination arms), containing a centromeric sequence (CEN), autonomously replicating sequence (ARS), and an auxotrophic element for selection and maintenance in yeast (Trp) (Fig. 4A). As we previously hypothesized that JG024 is circular permuted, terminal ends should be different on each copy of the JG024 genome. We used *in vitro* cleavage using SpCas9-sgRNA to target and cleave a precise location (target used: ACAATCCTCATAAGAAGTCGCGG) and obtain phage molecules linearized at the same position. After transformation, we obtained several hundred yeast colonies (Fig. 4B) and screened 10 clones. We first validated the presence of phage DNA using a unique PCR amplifying 827 bp of the JG024 genome and six yeast clones of the 10 screened showed amplification (Fig. S3A). We next validated the recombination event by amplifying recombination scars (Fig. S3B). Finally, for the presence of a full phage molecule, we performed multiplex PCR on 10 JG024 parts (Fig. 4D). Of the three screened clones, all were validated as containing a circular JG024 genome. In addition to full size JG024 DNA, we also used a second sgRNA to cut the genome simultaneously in a second genome location (target used 2: CTAGTGTACGCTAGAATCAGTGG) and clone the JG024 genome in two parts. We again used two recombination templates flanked by 60 bp of homology (recombination arms) specific for each JG024 fragment. For the first half, only one yeast clone out of 50 screened contained the expected phage DNA (Fig. 4B). In contrast, despite using the same JG024 DNA preparation for cloning and only different recombination arms, we obtained 8 clones of 10 screened that contained the second half. These results suggest that the TAR cloning efficiency is not uniform and may be impacted by the recombination arms, the size of the product to be cloned, and/or the nature of the product itself, among other potential factors.

**Fig 5:**
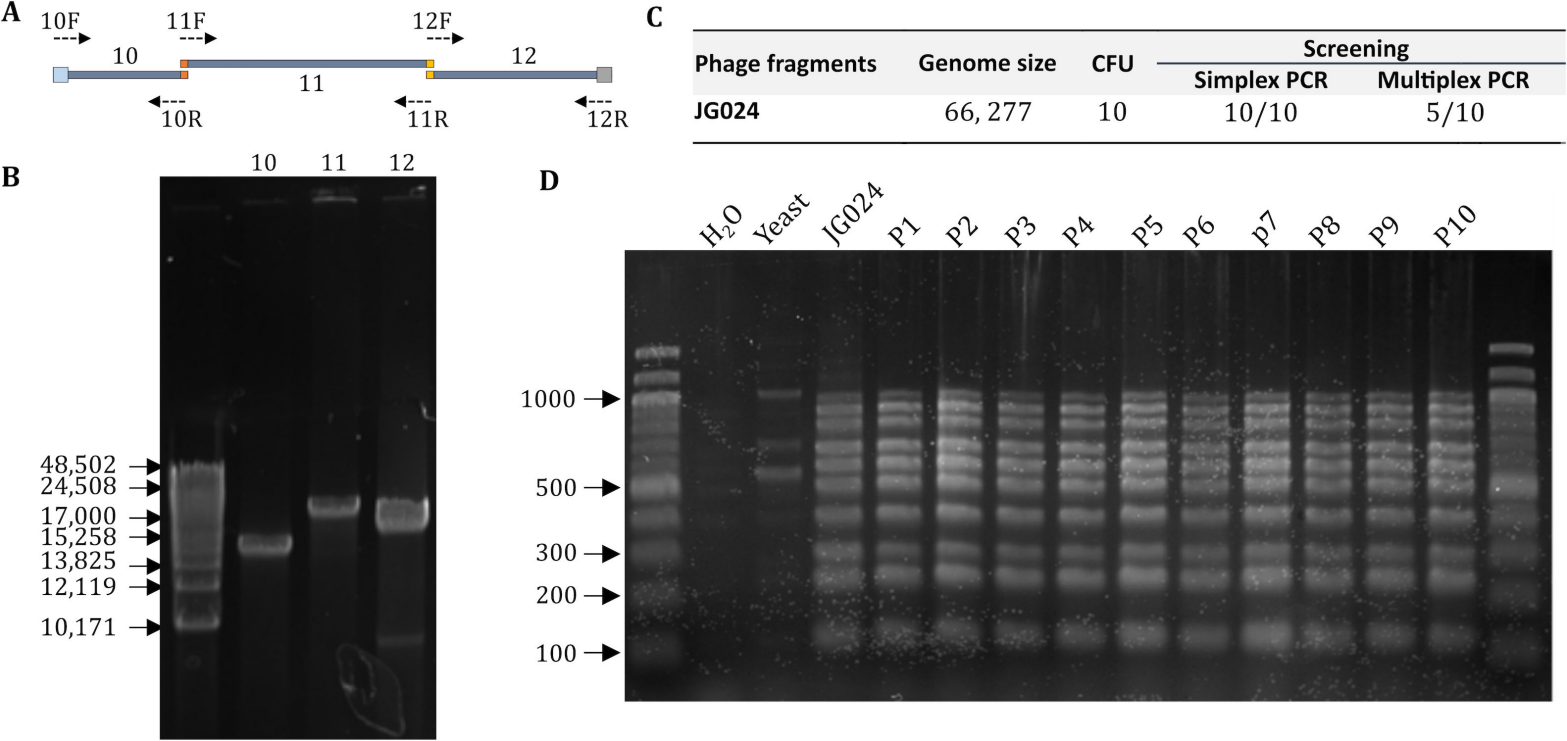
Construction of synthetic phage DNA in yeast. (A) Visual representation of the three-fragment PCR design for JG024 genome amplification. (B) Agarose gel of PCR fragments (10, 11, and 12 in panel A) obtained for JG024 cloning in yeast. (C) Cloning efficiency of assembled JG024 fragments in yeast. Simplex and multiplex PCR are as described in Fig. 4. (D) Agarose gel of multiplex PCR (as described in Fig. 4) of the JG024 genome obtained successively after 10 passages in yeast demonstrating the stability of the construct.

The eventual goal of this methodology is to permit the reboot of genetically engineered phage. To that end, we anticipate it may be desirable to clone a genome in multiple fragments, e.g., two ends of the WT genome surrounding a synthetic middle fragment, which could then recombine into a chimeric, edited genome in yeast. To determine whether this yeast strategy permits such genomic manipulation, we attempted to synthetically reconstruct the JG024 genome from multiple PCR fragments. From phage DNA, we amplified the JG024 genome in three overlapping DNA fragments (Fig. 5A and B) using primer sets primer4-6.F and primer 4-6.R. After transformation in yeast, we obtained 10 yeast colonies (Fig. 5C), which is a relatively low number of colonies compared to TAR cloning (>350; Fig. 4B). However, as the assembly requires more recombination events than TAR cloning an individual molecule, increasing the recombination arm’s length could improve the number of transformants. Despite this low colony number, we obtained 5/10 clones with full-sized JG024 genomes.

DNA stability over time is critical for maintaining and performing genome engineering in yeast. To test the stability of the synthetic JG024 genome in yeast, we performed 10 successive passages and observed the DNA integrity using multiplex PCR (Fig. 4D). After 10 passages, we did not observe any DNA rearrangement and we thus concluded that JG024 phage DNA is stable in yeast. In summary, we successfully cloned the JG024 genome in yeast directly from extracted phage genomic DNA. We further demonstrate the simultaneous use of two sgRNA for JG024 modification purposes. We also showed that synthetic DNA could be used for the construction of JG024 genomes with large DNA modifications.

### Unsuccessful cloning of JG024 and smaller fragments in *E. coli*

Manipulation of cloned phage genomes in *E. coli* would be convenient to avoid limitations related to working in yeast, e.g., the small yield of cloned product relative to the yeast genome size. To enable downstream cloning in *E. coli*, we used a recombination template that contained not only the previously described yeast element but also an *E. coli* element (OriV, chloramphenicol acetyltransferase gene). However, we only observed colonies (*n* = 7) in one of the three replicates. Of those, only two were able to grow in liquid culture, and none showed the presence of JG024 DNA. We further attempted to clone the halved JG024 genome in *E*. *coli*, but no colonies were obtained for either half after three attempts at transformation. These results suggest that the size of the JG024 genome alone is not solely responsible for its toxicity in *E. coli*. Additional contributing factors may include a lysis protein encoded on the JG024 genome or other toxic elements, e.g., those inhibiting host DNA replication (27). Genome manipulation in yeast and reboot in a suitable host is thus not a matter of preference but rather of necessity.

### Identification of phage- and host-specific limits to phage reboot

To reboot JG024 DNA from the yeast clones, we extracted DNA and first attempted to transform PA using 10 µg of yeast DNA extraction. However, no plaques were observed in either PA14 or PAO1 strains. We hypothesized that factors related to JG024 itself, bacterial factors in the strain that is used for rebooting, or some combination of the two were inhibiting the reboot of the synthetic JG024 construct.

**Fig 6:**
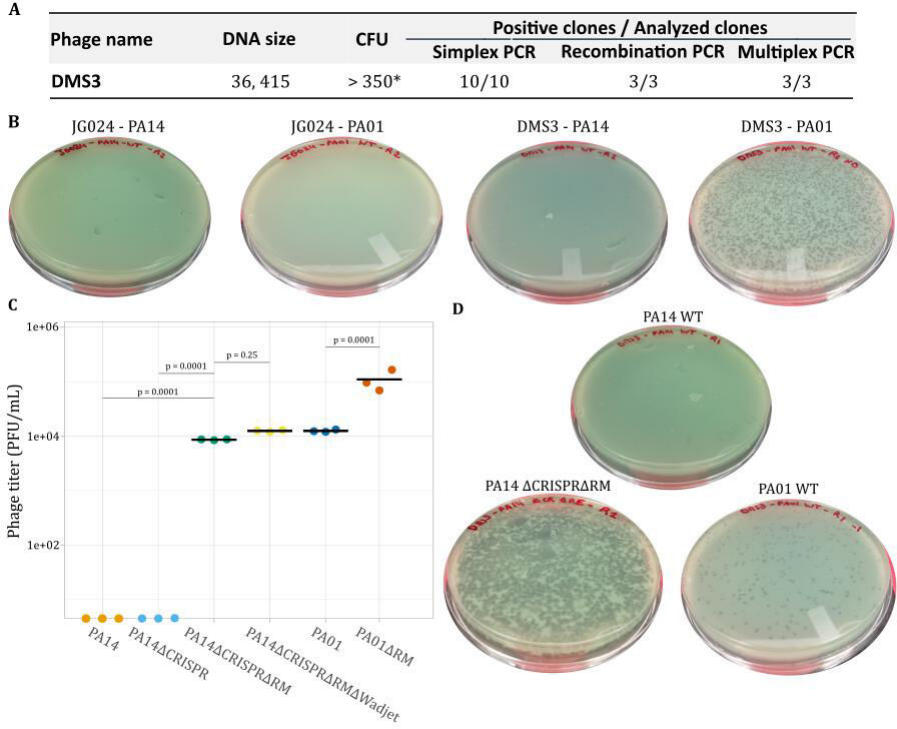
Comparison of DMS3 reboot to JG024 in PA14 and PAO1. (A) Cloning efficiency of DMS3 in yeast. Simplex PCR consists of one PCR that amplifies a single region of the genome. Recombination PCR involves amplification of recombination scars, and multiplex PCR uses a set of several primers to amplify multiple regions around the phage genome (in this case, six). (B) Example reboot result from yeast DNA obtained for linearized DMS3 and JG024 genomes in PA14 and PAO1. (C) Reboot of linear DMS3 phage DNA from yeast in wild-type PA14 and PAO1, as well as PA mutants lacking CRISPR (∆CRISPR), restriction-modification (∆RE), and Wadjet (∆Wadjet) defense systems. The knockout strains had a significant effect on the phage titer (analysis of variance; *P* = 0.007). The PAO1∆RE strain had a 42-fold higher phage titer than the PAO1 WT strain (*t*-test; *P* = 0.0001). Individual *P*-values represent the results of *t*-tests between incremental defense system removals (e.g., ∆CRISPR and ∆CRISPR∆RM). There was no significant difference in phage titer between PA14 and PA14∆CRISPR. (D) Example reboot result using linearized DMS3 genomes from yeast in PAO1, PA14, and a PA14 mutant lacking CRISPR and restriction-modification defense systems.

To understand if the synthetic JG024 genomic construct itself is problematic for rebooting purposes, we attempted to replicate our observations with another phage. For comparison, we selected DMS3 (48–50), which is part of the Casadabanvirus family of phages. Similar to JG024, the genome of DMS3 is predicted to be circular permuted DNA (51). In addition, the DMS3 genome naturally encodes anti-CRISPR and anti-quorum-sensing proteins (51). The genome of DMS3, at 36 kb, is also substantially smaller than that of JG024. We cloned DMS3 DNA in yeast using TAR cloning and validated genome integrity as described for JG024 (Fig. 6A). We next tried to transform synthetic DMS3 genomes in PA14 and PAO1. In contrast to JG024 (Fig. 6B), we observed DMS3 plaques but only in the PAO1 strain. We corroborate our previous findings that strain-level differences in hosts (PA14 or PAO1) impede or enhance reboot. Finally, we validate that DMS3 phage can be rebooted from a genome generated in yeast, which further suggests that phage-specific characteristics also impact reboot success.

We next tried to understand why the DMS3 phage could be rebooted in PA01 but not in PA14, and JG024 could not be rebooted in either. Multiple determinants are responsible for bacterial strain specificity, including differences in receptors, superinfection immunity or exclusion, and differences in antiviral defense systems (52, 53). As wild-type JG024 infects both PA14 and PAO1, we do not expect differences in the expression of lipopolysaccharide (JG024 receptor) or superinfection to be major barriers to reboot. We thus hypothesized that the difference observed in reboot between PA14 and PAO1 could be linked to their antiviral defense systems. We identified these systems using PADLOC (Table S4) (54) and found at least four that could interact with DNA and impact reboot: a type-I RM system in PAO1, and type-II RM, type I CRISPR, and Wadjet systems in PA14. RM systems protect endogenous DNA and cleave exogenous DNA via methylation discrimination. Production of phage genome in yeast will affect its DNA methylation profile and could be a limitation to DNA transformation and phage reboot. Type I-F CRISPR system and Wadjet systems are composed of several proteins that possess nuclease activity and could then interact with phage exogenous DNA (49, 55), preventing DNA transformation and phage reboot. To determine if host antiviral systems were inhibiting phage rebooting from phage genome cloned in yeast, we used four PA mutant strains: PAO1∆RE, PA14∆CRISPR, PA14∆CRISPR∆RE, and PA14∆CRISPR∆RE∆Wadjet, validated by whole-genome sequencing (Table S3). Plasmid DNA transformation efficiency was similar between our WT strains and mutants (*P* > 0.05) (Fig. S5). Finally, we tried to reboot DMS3 phages using linear DNA from yeast extractions. As observed previously (Fig. 6B), DMS3 reboot was not observed in either PA14 or PA14∆CRISPR (Fig. 6D). In contrast, when the type-II RM system was removed, we observed a consistent reboot (*P* = 0.0001) (Fig. 6D). Additional removal of the Wadjet system resulted in a 1.4-fold increase in plaque numbers; however, this improvement was not statistically significant (*P* = 0.25). In PA01, the reboot of the linearized synthetic DMS3 construct was previously successful in WT PAO1 (Fig. 6B), but the removal of the type-I RM system resulted in an 8.8-fold increase in plaques (*P* = 0.0001) (Fig. 6D).

**Fig 7:**
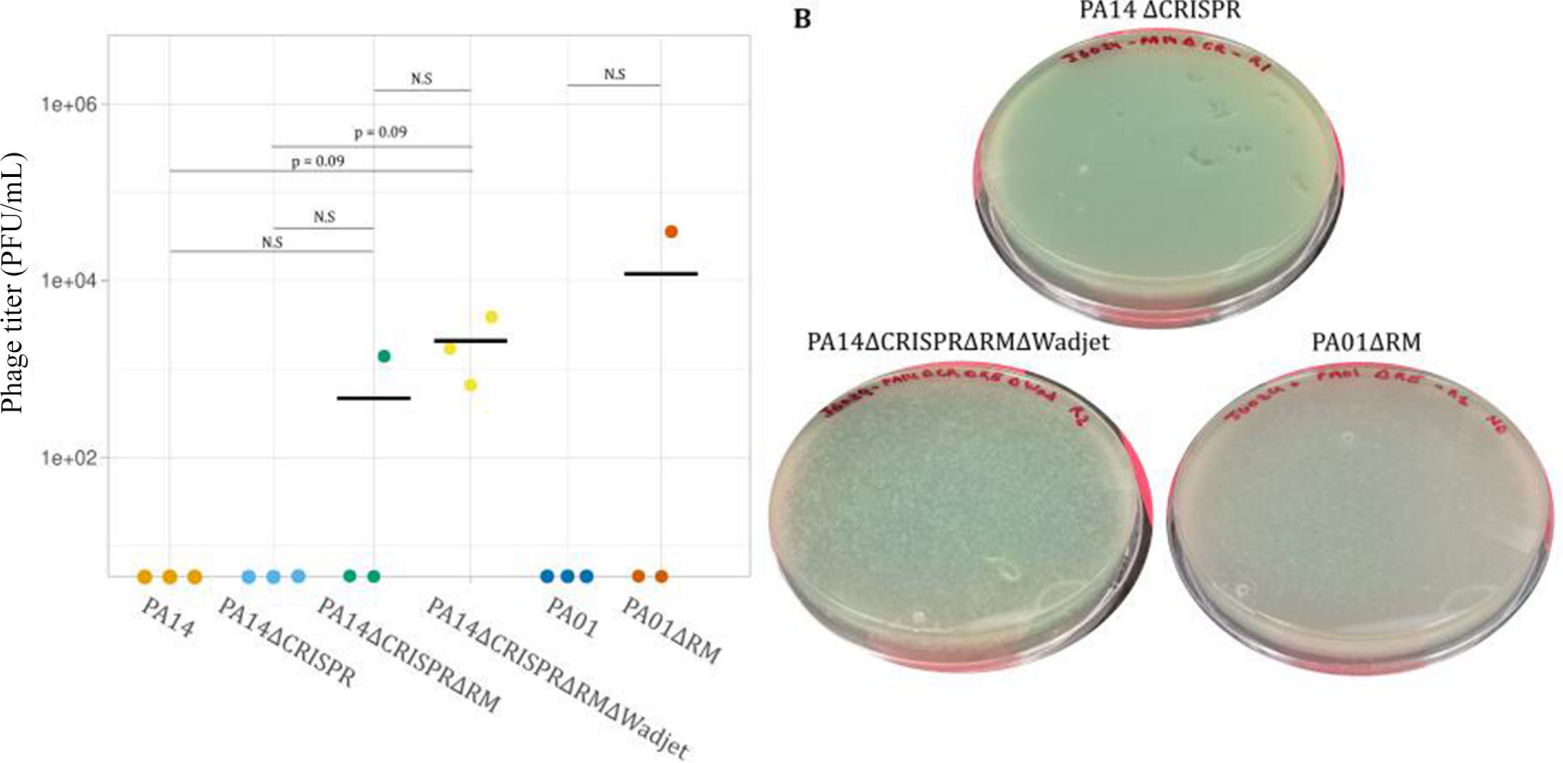
Phage reboot from yeast DNA in PA mutants. (A) Reboot of linearized JG024 phage DNA from yeast in wild-type PA14 and PAO1, as well as PA mutants lacking CRISPR (∆CRISPR), restriction-modification (∆RE), and Wadjet (∆Wadjet) defense systems. Individual *P*-values represent the results of *t*-tests between incremental defense system removals (e.g., ∆CRISPR and ∆CRISPR∆RM). N.S, not significant (*P* > 0.05). (B) Example reboot result of linearized JG024 genomes obtained from yeast in PA14 and PAO1 mutants, denoted as in panel A.

Next, we tried to reboot JG024 in the PA defense system knockouts. We did not obtain rebooted phage using PA14 or PA14∆CRISPR (Fig. 7) as observed for DMS3 phage, nor were we able to reboot using PAO1 as previously observed (Fig. 6B). However, removing the type-II RM system from PA14-enabled phage reboot, albeit only in one out of three replicates (Fig. 7). When we removed the Wadjet system, we observed a more consistent reboot with replicable results in comparison to the type-II RM mutant (Fig. 7). Finally, in PAO1, we also obtained reboot with JG024 DNA when the type-I RM was removed (Fig. 7). If we compare JG024 and DMS3 reboot (Fig. 6C and 7), JG024 is less efficiently rebootable compared to DMS3 as we used 2.5 h of rebooting time for DMS3 instead of 6 h for JG024, but we still observed more efficient reboot for DMS3. In summary, in addition to genome circularity (or removal of the yeast element), we observed that defense systems can interact with phage DNA produced in yeast. In particular, the PA14 type-II RM system can lead to total inhibition of phage reboot, whereas other systems may dampen efficiency or decrease repeatability. Finally, phage-defense system interactions are specific to the phage and host in question.

### Phage genome validation by whole-genome sequencing

Finally, we investigated whether the methodology created mutations in rebooted phages by performing whole-genome sequencing on the wild-type DMS3 and JG024 phages and four clones of rebooted phages (two DMS3 reboots and two JG024 reboots) using the Illumina NextSeq 2000 platform (Table S5; Fig. S5). We expected two different types of mutations: stochastic mutations that appear during the phage replication process, which would be present unevenly across reads, and mutations linked to the methodology, which could be generated in yeast or during the cloning process. The latter should be represented as an ancestral mutation and thus be present on the overwhelming majority of reads, as a phage plaque is generated from a single phage that was generated in a single reboot event using a single copy of phage DNA produced in yeast. Comparing to the reference genome, we detected only seven low-frequency mutations (not related to the methodology) in the two JG024 rebooted clones (details in Supplemental Results) and no high-frequency mutations except the two already present in the WT. In the two rebooted DMS3 clones, we detected 13 low-frequency mutations (details in Supplemental Results) and eight high-frequency mutations, of which seven were already present in the WT and only one was newly found in the two rebooted clones (position 36,389, C to T). This last mutation is likely linked to the methodology since the site of this mutation is on a recombination arm used to add yeast elements during the cloning step. It is possible that the mutation was introduced during PCR amplification of the recombination arm. Finally, we conclude that the methodology has high fidelity with minimal introduction of mutations.

### Expanding the methodology to other phages

To begin to explore how generalizable this method is, we tried to reboot two additional phages: vB_PaeP_PAO1_Ab05 (56), member of the Autographiviridae family (*Phikmvvirus* genus) and F8 phage (57), a member of the *Pbunavirus* genus. In comparison with DMS3 and JG024, vB_PaeP_PAO1_Ab05 has a different genome structure with DTR (431 bp), and both vB_PaeP_PAO1_Ab05 and F8 are able to infect PAO1 but not PA14. We first successfully cloned vB_PaeP_PAO1_Ab05 and F8 genomes in yeast (Fig. S6). Despite the inability of either phage to infect PA14, we successfully rebooted both in the triple-mutant strain PA14∆CRISPR∆RE∆Wadjet. This suggests that the methodology can be used to reboot diverse phages, even if the phage is not able to infect PA14.

## DISCUSSION

Using JG024, we developed a methodology for the construction of tailed PA phages, which are promising for phage therapy applications. This is the first step toward constructing “à la carte” phage genomes with specific traits and characteristics. Our analysis of JG024 has improved our understanding of this phage, particularly regarding the genome structure, with evidence indicating a circular permuted genome. The use of yeast as a platform for cloning and assembling phage genomes is an important step in advancing methodology for genomic manipulation of diverse phages, which must be coupled to a robust reboot strategy. We identified three major limitations to reboot from phage genomes cloned in yeast. By cloning and rebooting DMS3, another tailed phage, we demonstrated that different phage species have different reboot efficiencies. We identified bacterial defense systems that inhibit phage reboot from genomes cloned in yeast. Finally, we demonstrate the possibility to reboot two more PA phages (vB_PaeP_PAO1_Ab05 and F8) that are not able to infect the strain used for reboot. Together, as a proof of concept, we demonstrate the possibility to reboot PA phages that belong to the three families of phages (Podoviridae, Siphoviridae, and Myoviridae) and we identified barriers to the construction of synthetic, clinically relevant phages.

In general, knowing the genome structure can influence the design of cloning and manipulation in yeast. For example, terminal ends could restrict the possible insertion sites for a yeast element. Our study suggests that the JG024 genome is circular permuted. Unicycler and Flye assembly suggest a circular genome in contrast to Trycycler assembly and Phageterm analysis. However, as Nextera transposon-based library preparation was used to prepare Illumina short-read sequencing data, it was expected that phage termini would not be detectable by methods such as PhageTerm because transposome sequence bias would likely misrepresent the distribution of read edge positions that are necessary for terminus prediction (58, 59). For example, Chung et al. (59) were unable to identify the termini of the novel *Bacillus cereus* phage SBP8a using Nextera-derived MiSeq sequencing data but identified a DTR of 2,821 nt with Roche/454 sequencing data. Thus, the biased nucleotide frequency of the Nextera-derived reads may have altered the distribution of read edge positions to produce artificially high coverage regions, which were detected by PhageTerm as DTRs in this study. Indeed, Sanger sequencing results conflict with the DTR predicted by Trycycler and PhageTerm. Furthermore, the successful amplification of overlapping fragments that cover the full JG024 genome, the digestion profile, and the mapping of long-read sequencing data (>30,000 bp) suggests that JG024 has a circular permuted genome. Experimental verification, e.g., by Southern blot analysis (41), is needed to make this observation conclusive. Further experiments could also verify the headful packaging strategy with a putative packaging site at position 59,376 bp.

Other characteristics, primarily related to transformation efficiency, are important for ensuring a successful reboot (Fig. 3; Fig. S2). As identified for at least 30% of tailed phage (43), we determined that JG024 is sensitive to chloroform. This is particularly important for experimental design as chloroform is used to release phage particles from bacterial cells for many types of phage experiments (24, 60). We also worked on transformation parameters that were already developed (61, 62) to obtain an optimized protocol for the reboot process for JG024 (Fig. 3; Fig. S2). As this type of work expands, additional data will become available for a more diverse phage. This will, in turn, enable generalized conclusions about the information needed to design and optimize a reboot protocol for any given phage.

Yeast has been extensively used as a cloning platform for high-length DNA molecules since 1980 (63). Different methods have been developed to clone genomic fragments and full-length virus or bacterial genomes in yeast (45, 64–67), and each of these methods requires the addition of yeast elements (Ars, Cen, and Trp) to maintain the DNA molecule in yeast over time. These methods have allowed the cloning of genomes up to 1.8 Mb (28, 65), and as expected for small phage genomes, we successfully obtained several yeast clones containing stable JG024 genome and DMS3 genomes (Fig. 3 and 4). We used TAR cloning (64) and genome assembly (67) methods to construct JG024 DNA, and those methods opened up numerous possibilities for genome engineering during the cloning step. Furthermore, the yeast platform has the advantage of allowing the cloning of phage cargo genes that would be toxic for *E. coli* (68). In contrast to *E. coli* machinery that can recognize and express many prokaryotic genes (68, 69), the yeast machinery, which is eukaryotic, is unlikely to express prokaryotic genes, as most of the transcription signals are not recognized (31). This is particularly interesting for the cloning of phage genomes, which often contain genes for toxic proteins, such as toxin-antitoxin systems for phage selection pressure (70) or endolysin for phage release (71, 72). As observed in our study, JG024 genome cloning in *E. coli* was not functional. As *P. aeruginosa* and *E. coli* are closely related, we hypothesize that some JG024 genes can be expressed in *E. coli* and are toxic for the bacterial cell, but this still needs further experimental verification.

The yeast platform can also have several disadvantages. For example, as homologous recombination is efficient in yeast, even with the presence of yeast elements (ARS-CEN-Trp), DNA instability could occur through small DNA repeat sequences that can recombine and generate truncated versions of the genome over time (73). Our data showed that for JG204 phage, the genome is stable over 10 passages (Fig. 4). The genome structure of phage containing DTRs could generate instability, but our results with vB_PaeP_PAO1_Ab05, in addition to a recent paper on *S. aureus* phages containing DTRs (25), suggest that this is unlikely to be a widespread issue.

Another issue that we identified is the DNA methylation profile of the phage genome after production in yeast. DNA methylation in yeast is rare (74, 75), which is problematic for the use of this DNA to transform some bacterial strains. For example, it has already been described that for bacterial transplantation from genomes cloned in yeast, it was necessary to remove RM systems from the bacterial host strain or to perform *in vitro* methylation using cell extracts from the bacterial host strain (29). Indeed, the synthetic phage genome constructed in yeast is likely unmethylated and thus a target for cleavage by an RM system. PA possesses multiple antiviral defense systems, including CRISPR (76) and RM systems, and strains PAO1 and PA14 are no exceptions (Table S4). Our data confirm that RM can be problematic for DNA transformation from yeast DNA, particularly in PA14 where no phage reboot was observed in the presence of type-II RM genes (Fig. 5D and 6A). We also see that the type-I RM system from PAO1, while not completely inhibiting DMS3 phage reboot, decreases the reboot efficacy (Fig. 5D). This shows that different types of defense systems, in particular RM systems, will have different impacts on phage reboot, and removing those systems increases the probability of success. Several other defense systems have been identified in PAO1 and PA14, of which Wadjet systems are particularly notable. These defense systems, recently described in *Bacillus subtilis* and *P. aeruginosa*, recognize and cut DNA based on its topology, resulting in reduced transformation efficiency in *B. subtilis* (55, 77). Another study showed that Wadjet JetABCD systems restrict circular plasmids in *B. subtilis* but a linear plasmid evades restriction by *E. coli* JetABCD *in vivo* (78). When removing the Wadjet system from PA14, we did not observe an increase in plasmid transformation efficiency (Fig. S4A), but we observed a potential implication of the Wadjet system on the reboot consistency from the JG024 DNA genome previously cloned in yeast, with replicable results obtained using the strains without Wadjet system (Fig. 6A). Several experiments are needed to understand the exact implications of Wadjet systems on phage reboot in yeast-cloned genomes and to describe the molecular mechanism of Wadjet restriction in *P. aeruginosa*.

The use of yeast as a platform requires the use of a yeast element for circularization and maintenance of the phage genome in yeast. It is unclear if the presence of the yeast element on the phage genome can be problematic for subsequent reboots (see Supplemental Results). Previous phage reboot papers that use yeast or *E. coli* as a manipulation platform do not describe the release of the yeast element before phage reboot (23–25, 32, 79). However, *in vitro* genome assembly has been used to demonstrate that DNA circularity increased reboot efficiency (79). Using our reboot conditions, JG024 was not able to reboot as a circular molecule (see Supplemental Results). This is possibly due to DNA length, which is higher by 9.3 kb when containing yeast and *E. coli* elements, or topology rather than DNA circularity. More experiments are needed to understand this phenomenon and whether it impacts other phage reboot methodologies.

Finally, in this work, we have developed a method for rebooting clinically relevant *P. aeruginosa* phage. This is the first step toward important genome engineering that could be performed on JG024, DMS3, and other phages to improve and add specific phenotypic traits that could be useful for phage therapy applications. For example, changing the receptor to, e.g., expand the host range of PA strains that could be infected (24), adding anti-CRISPR proteins to prevent CRISPR adaptation by the targeted PA strain (21) or adding anti-quorum-sensing proteins to inhibit biofilm production (51). This work thus represents a critical step towards using phage therapy to overcome antimicrobial resistance and treat infection.

## MATERIALS AND METHODS

### Oligonucleotides and plasmids

All oligonucleotides used in this study were supplied by Integrated DNA (IDT) and are described in Table S1. All plasmids constructed and used in this study are listed in Table S2.

### Microbial strains and culture

*Pseudomonas* phage DSM 19871 (JG024) (39) was obtained from DSMZ (Braunschweig, Germany). Phage DMS3 (48), *Pseudomonas aeruginosa* PAO1 (Tax ID: NC_002516), and PA14ΔCRISPR (49) were provided by Prof. George A. O’Toole (Geisel School of Medicine at Dartmouth). Additional *P. aeruginosa* mutants were constructed as described in the supplemental material. *P. aeruginosa* strains were cultivated at 37°C in lysogeny broth (LB) media or Vogel-Bonner minimal medium. Gentamycin at 50 µg mL^−1^ or carbenicillin at 300 µg mL^−1^ were used for selection. F8 phage (57) and vB_PaeP_PAO1_Ab05 phage (56) were provided by Prof. Joseph Bondy-Denomy.

*S. cerevisiae* VL648-N was provided by Dr. Carole Lartigue (INRAE). *S. cerevisiae* MAV203 (Thermo Scientific, 11445012) and VL648-N were cultured in yeast peptone dextrose adenine (YPDA; Takara, 630464) or SD-Trp Broth (Takara, 630411 and 630413) at 30°C with shaking at 225 rpm.

### *S. cerevisiae* VL648-N transformation procedure

Phage genome cloning in non-commercial VL648-N strain was performed following references (47, 64) and (45) with several modifications. For cloning half genome of JG024, *in vitro* cleavage of phage DNA was performed using the *Streptococcus pyogenes* CRISPR system. sgRNA was produced using EnGen sgRNA Synthesis Kit (NEB, E3322S), primer D31 or D32, and purified using Monarch RNA Cleanup Kit (NEB, T2040S). Cas9 nuclease (NEB, M0386S), sgRNA and 1 µg of phage DNA were incubated at 37°C for 20 min. Cas9 was then inactivated by incubation at 65°C for 10 min.

### Yeast DNA extraction

Individual yeast colonies were picked and streaked on SD-Trp and incubated for 2 days at 30°C. Then, one isolated colony per streak was patched on SD-Trp plate and incubated for 2 days at 30°C. Total genomic DNA was extracted from yeast transformants according to reference (64).

### Phage reboot protocol

Phage reboot was performed using a previously described PA electroporation protocol with some modifications. Different parameters were tried as described in the Results section. Finally, MgSO_4_ buffer was used for washing cells, 100 ng of phage DNA was used as a control, LB was complemented with 1 mM MgSO_4_, and incubation of 3–24 h was performed for cell regeneration and phage production. For chloroform assays, two to three drops of chloroform were added to the cell suspension after incubation to kill bacterial cells and release the phages. For reboot from yeast DNA, separation and release of a linear phage DNA from the yeast recombination matrix were performed using 10 µg of yeast DNA digested using SmaI (NEB, R0141S) for JG024 and ScaI (NEB, R3122S) for DMS3, vB_PaeP_PAO1_Ab05, and F8. Restriction enzymes were inactivated by 80°C heat inactivation for 20 min, and DNA was then kept at 4˚C until transformation.

To quantify PFUs after phage reboot incubation, serial dilutions of supernatant were made with LB media. Three hundred microliters of supernatant was separately mixed with 200 µL of mid-exponential PA14 cells and 4 mL of LB soft agar (0.8%) complemented with 1 mM MgSO_4_ and prewarmed to 55°C. The agar mixture was then poured onto LB plates and incubated overnight at 37°C. The plates containing phage plaques were then counted.

### Statistical analysis

To determine the significance of the main parameter effects on PFUs, an analysis of variance was performed on the log-transformed data at an α level of 0.05 using the statistical software JMP Pro 16.0.0 (SAS Institute Inc., Cary, NC, USA). *Post hoc* multiple comparisons were conducted using Tukey’s HSD tests.

### Short- and long read sequencing

Library preparation, short- and long-read sequencing (Illumina and Oxford Nanopore technologies, respectively), and *de novo* assembly were performed by the Microbial Genome Sequencing Center (MiGS; Pittsburgh, PA, USA). For DMS3 and JG024 phage reboot clone and PA14 and PAO1 host defense system deletion verification, Illumina NextSeq 2000 sequencing was performed, presented in Table S5. Illumina paired-end reads (2 × 151 bp) were obtained using the Illumina DNA Prep Kit, IDT 10 bp UDI indices, and the Illumina NextSeq 2000 platform (80). Demultiplexing, quality control, and adapter trimming were performed by MiGS with bcl-convert (v4.0.3). Quality control was checked using FastQC (v0.11.5) (https://www.bioinformatics.babraham.ac.uk/projects/fastqc/) and MultiQC (v1.11).

ONT sequencing libraries for JG024 WT were prepared using Oxford Nanopore’s “Genomic DNA by Ligation” kit (Oxford Nanopore Technologies, Oxford, UK) and sequenced on a MinION R9 flow cell. Base calling for ONT long reads was performed using Guppy HAC basecalling mode (v4.2.2) (81). bcl2fastq v2.20.0.445 (82) and Porechop v0.2.3_seqan2.1.1 (83) were used for quality control and adapter trimming for Illumina and ONT sequencing, respectively.

### Hybrid assembly of JG024 WT

Initial hybrid assembly of JG024 WT was conducted by MiGs via Unicycler v0.4.8 and yielded one circular contig (66,277 bp; GC content: 56%). In addition, ONT long reads were then filtered using Filtlong (v0.2.1) (--keep_percent 95) (84) and assembled with Trycycler (v0.5.3) (85) using Raven (v1.7.0) (86), Flye (v2.9-b1768) (87), and miniasm (v0.3-r179) (88) to yield one linear and one circular contig. These contigs were then polished using Medaka (v1.3.2) (https://github.com/nanoporetech/medaka). Illumina short reads were then used to further polish each contig using polypolish (v0.5.0) (89) and POLCA (from MaSuRCA v4.0.7) (90) for two rounds each to yield one linear (66,307 bp; GC content: 56%) and one circular (66,277 bp; GC content: 56%) final contigs. The phage genome termini of the circular contig were predicted using PhageTerm (58), and the quality of the overall assemblies was assessed with CheckV (91). For Fig. 2C observation, Nanopore reads were filtered using Filter FASTQ (V 1.1.5, minimum size 30,000 bp), mapped using BWA-MEM (V 0.7.17.1), and visualized on IGV (92).

### Host deletion and phage reboot sequencing analysis

For *P. aeruginosa* strain verifications, analyses were made using Galaxy (https://usegalaxy.eu/). Illumina reads were trimmed using Trimmomatic (V 0.38.1; Sliding Window 10, 20; Drop read below Minimal length 150), mapped using BWA-MEM (V 0.7.17.1), Samtools sort (V 2.0.3), and MPileup (V 2.1.1), and variants were detected using VarScan mpileup (V 2.4.3.1; Minimum coverage 20, Minimum supporting read 15, Minimum Base quality 20, Minimum variant allele frequency 0.5, and Minimum homozygous variants 0.75). For defense system mutants, deletions were verified using JBrowse (V 1.16.11).

To detect mutations in the rebooted phage, read mapping was performed using BWA-MEM (v0.7.12) with default parameters. DMS3 and JG024 WT and clones were mapped to their NCBI reference genomes, NC_008717 and NC_017674, respectively. Mapped reads were converted to BAM format using the Samtools (v1.6) view command, sorted using the sort command, and reads were piled using the mpileup command. Indels and single nucleotide polymorphisms were identified using VarScan (v2.4.6) set to a --min-coverage=30, --min-reads2=20, --min-var-freq=0.01, and --min-freq-for-hom=0.75. All other VarScan parameters were run as default. VCF outputs from VarScan were visualized using IGV (v.2.8.10). Specific SNP and indel locations were compared with reference genome annotations on NCBI.

## Data Availability

All sequencing data are available via the NCBI Sequence Read Archive (BioProject: PRJNA1019263).

## SUPPLEMENTAL MATERIAL

Table S1 spectrum.02897-23-s0001.xlsx List of oligos.

Table S2 spectrum.02897-23-s0002.xlsx List of plasmids.

Table S3 spectrum.02897-23-s0003.xlsx List of bacterial strains.

Table S4 spectrum.02897-23-s0004.xlsx Detection of defense systems.

Table S5 spectrum.02897-23-s0005.xlsx Verification of mutations in genomes by WGS.

Supplemental figures and text spectrum.02897-23-s0006.docx Supplemental figures, methods, and results.

ASM does not own the copyrights to Supplemental Material that may be linked to, or accessed through, an article. The authors have granted ASM a non-exclusive, world-wide license to publish the Supplemental Material files. Please contact the corresponding author directly for reuse.
