# Correction for Ipoutcha et al., “A synthetic biology approach to assemble and reboot clinically relevant *Pseudomonas aeruginosa* tailed phages”

**DOI:** 10.1128/spectrum.00322-25

**Published:** 2026-01-29

**Authors:** Thomas Ipoutcha, Ratanachat Racharaks, Stefanie Huttelmaier, Cole J. Wilson, Egon A. Ozer, Erica M. Hartmann

## AUTHOR CORRECTION

Volume 12, no. 3, e02897-23, 2024, https://doi.org/10.1128/spectrum.02897-23. Pages 9, 10, 11, and 14: The DMS3 bacteriophage used during the reboot experiments was incorrectly reported as wild type. It was a double knockout mutant: the first deletion is 227 bp in length from positions 500 to 727; the second deletion is 188 bp in length from positions 18400 to 18588.

